# The complete mitogenome of the Eastern Pacific sponge *Aplysina gerardogreeni* (Demospongiae, Verongida, Aplysinidae)

**DOI:** 10.1080/23802359.2019.1643804

**Published:** 2019-07-22

**Authors:** Manuel Ricardo Salas-Castañeda, Ana Castillo-Páez, Axayácatl Rocha-Olivares, José Antonio Cruz-Barraza

**Affiliations:** a Systematics and Molecular Ecology Laboratory, Universidad Nacional Autónoma de México, Instituto de Ciencias del Mar y Limnología, Unidad Académica de Mazatlán, Sinaloa, Mexico;; b Molecular Ecology Laboratory, Biological Oceanography Department, CICESE, Baja California, Mexico

**Keywords:** Porifera, Verongimorpha, Mexican Pacific Ocean, mitochondria

## Abstract

We report the first mitochondrial genome of a Verongid sponge, *Aplysina gerardogreeni* from the Pacific Ocean. This has 19,620 bp and includes 14 protein-coding genes, 2 rRNAs genes, and 25 tRNAs genes. The gene arrangement was similar to the one found in two Caribbean *Aplysina* mitogenomes previously reported. Comparative analyses revealed a few substitutions among congeneric mitogenomes. The mitogenome of *A. gerardogreeni* could be useful to study the evolution of Verongimorpha group and also to identify adequate genes for its molecular systematics.

Sponges have become increasingly relevant in evolutionary, ecological, economical, and biotechnological (e.g. pharmaceutical, biomaterial) research. Species of genus *Aplysina* are known for producing diverse metabolites with antibiotic and cytotoxic activities (Lira et al. 2011; Puyana et al. 2015). Probably, its principal importance comes from its chitin fiber skeleton, which has promising biomedical applications in tissue engineering (Ehrlich et al. 2007; Ehrlich et al. 2010). Despite its importance, the lack of diagnostic characters and the high phenotypic plasticity of some species greatly complicate its taxonomic identification, hindering its potential application (Cruz-Barraza et al. 2012).

*Aplysina gerardogreeni* is the most conspicuous Verongid sponge from the Eastern Pacific, found from Mexico to Panama (Caballero-George et al. 2010; Cruz-Barraza et al. 2012). Two specimens were collected from Mazatlán Bay, Mexico (23°15′29″N, 10°28′25″W) in August 2015 and deposited in the ‘Colección de Esponjas’ (OAX-MAM-135-10-02) of the Instituto de Ciencias del Mar y Limnología, UNAM (LEB-ICML-UNAM-3173). Genomic DNA was extracted from fresh tissue using WizardVR Genomic DNA Purification Kit (Promega, Madison, WI) following the manufacturer’s protocol. Separate DNA libraries were constructed with the Kapa gDNA library kit (Kapa Biosystems, Wilmington, MA) using multiplex index. They were sequenced using 2/7 a single lane (2 × 125 paired-end reads) in a MiSeq platform (Illumina, San Diego, CA). Quality control and trimming were implemented in CLC Genomics Workbench 7.0.3 (CLC bio, Boston, MA). Using published mitogenomes of *A. fulva* and *A. cauliformis*, we aligned about 70% of the *A. gerardogreeni* genome and designed new sets of primers to fill in the gaps by PCR amplification and Sanger sequencing. Gene identification was carried out by MITOS (Bernt et al. 2013) and by homology with other *Aplysina* mitogenomes.

The mitogenome of *A. gerardogreeni* (GenBank Accession: MN082378) was 19,620 bp in length, contained 2 rRNA genes, 25 tRNA genes (tRNA MET and tRNA SER1 were duplicated), and 14 protein-coding genes. The overall base composition was 34.1% Adenine, 33.1% Tymine, 15.5% Cytosine, and 17.3% Guanine, and 32.8 GC%. The mitochondrial gene arrangement of *A. gerardogreeni* was similar to the one found in Caribbean congeneric species. Mitogenome alignments revealed 71 variable sites with *A. cauliformis* and 58 with *A. fulva*. The low variability between genomes could be expected due to its slow-evolving in some early-splitting lineages as Porifera (Huang et al. 2008). The genes ND4, ND1, and ND2 showed more mutations than other protein-coding genes among the species of *Aplysina*.

Maximum likelihood phylogenetic trees were reconstructed with the nucleotides of 14 protein-coding genes (Figure 1) using MEGA X v.10.0.5 (Kumar et al. 2018) along with 10 Demospongidae mitogenomes from NCBI. We confirmed the monophyly of *Aplysina* species and the low variation among their mitogenomes.

**Figure 1.**
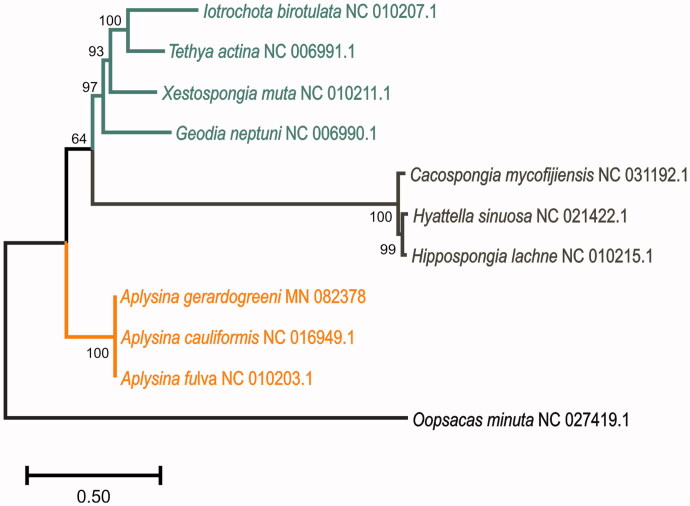
Maximum Likelihood tree obtained of concatenated nucleotide sequences of 14 mitochondrial protein-coding genes from *A. gerardogreeni* and other Demospongiae species, including Heteroscleromorpha (green) Keratosa (brown) and Verongimorpha (orange) Subclasses. *Oopsacas minuta* from Class Hexactinellida (black) was used as an outgroup (substitution model GTR + G, percentage of bootstrapping after 500 replicates).
